# Adoption and sustainability of decision support for patients facing health decisions: an implementation case study in nursing

**DOI:** 10.1186/1748-5908-1-17

**Published:** 2006-08-24

**Authors:** Dawn Stacey, Marie-Pascale Pomey, Annette M O'Connor, Ian D Graham

**Affiliations:** 1 School of Nursing, University of Ottawa, 451 Smyth Road, Ottawa, Canada; 2 Clinical Epidemiology Program, Ottawa Health Research Institute, 1053 Carling Avenue, Ottawa, Canada; 3 University of Montreal, Montreal, Canada

## Abstract

**Background:**

Effective interventions prepare patients for making values-sensitive health decisions by helping them become informed and clarifying their values for each of the options. However, patient decision support interventions have not been widely implemented and little is known about effective models for delivering them to patients. The purpose of this study was to describe call centre nurses' adoption of a decision support protocol into practice following exposure to an implementation intervention and to identify factors influencing sustainable nursing practice changes.

**Methods:**

Exploratory case study at a Canadian province-wide call centre guided by the Ottawa Model of Research Use. Data sources included a survey of nurses who participated in an implementation intervention (n = 31), 2 focus groups with nurses, interviews with 4 administrators, and a document review.

**Results:**

Twenty-five of 31 nurses responded to the survey measuring adoption of decision support in practice. Of the 25 nurses, 11 had used the decision support protocol in their practice within one month of the intervention. Twenty-two of the 25 intended to use it within the next three months. Although some nurses found it challenging to begin using the protocol, most nurses reported that it: a) helped them recognize callers needing decision support; b) changed their approach to handling these calls; and c) was a positive enhancement to their practice. Strategies identified to promote sustainability of practice changes included integration of the decision support protocol in the call centre database, streamlining the patient decision aids for easier use via telephone, clarifying the administrative direction for the call centre's program, creating a call length guideline specific for these calls, incorporating decision support training in the staff development plan, and informing the public of this enhanced service.

**Conclusion:**

Although most nurses adopted the decision support protocol for coaching callers facing values-sensitive decisions, to sustain practice changes, interventions are required to manage barriers in the practice environment and integrate decision support into the organization's policies, resources, and routine activities.

## Background

I was very excited about my pregnancy until I saw the doctor. She suggested that because I'm 37, I need to consider whether or not to have an amniocentesis and then gave me some information. Now it seems my life is turned upside down; one day I think I should have the amnio but the next day I don't want to risk losing the baby. I feel that I know the facts but I'm torn!

'Simulated caller' Sam, age 37

Over the last several years, there has been a shift towards an informed, values-based decision making model in which patients, like Sam, are more involved in the process [1-3]. However, many patients making health decisions experience decisional conflict (uncertainty) and require guidance in understanding the information about their available options and clarifying their associated values [4]. Evidence-based patient decision aids, used as adjuncts to practitioner consultation, increase patient participation in decision making and improve decision quality [5]. When nurse coaching of patients in preparation for discussing decisions with their practitioner was combined with patient decision aids, the cost-effectiveness of the combined intervention was greater than with either decision aids alone or usual care [6]. Nevertheless, decision support interventions have not been widely implemented and delivery models for decision support services need to be evaluated [7,8].

Health call centres with 24-hour public access to telephone consultation by nurses are becoming more common. These centres offer symptom triage, health information, and, in some cases, values-sensitive decision support [9]. High quality decision support to prepare patients for discussing values-sensitive health decisions with their practitioners, involves clarifying the decision, monitoring decisional conflict, tailoring decision support to patients' needs, and facilitating and evaluating progress in decision making [10,11]. However, the quality of decision support provided through nurse call centres is variable, with most nurses providing information alone without addressing decisional needs related to unclear values or inadequate support. Common barriers influencing nurses' provision of decision support include limited usability of patient decision aids via telephone, lack of a structured approach to guide nurses discussing decisional needs, nurses' limited knowledge, skills, and confidence in providing decision support, unclear program direction, pressure to minimize call length, and low public awareness of decision support services [12]. One trial showed that compared to the control group, nurses that participated in an implementation intervention (i.e., online autotutorial, skill-building workshop, decision support protocol, and performance feedback on calls with simulated patients) showed statistically significant improvements in their knowledge and provided better quality decision support to simulated patient callers, without increasing call length [12,13].

The aims of this study were to describe call centre nurses' adoption of the decision support protocol following an implementation intervention and identify the factors influencing sustainable nursing practice changes within the call centre workplace environment. Adoption, according to the Ottawa Model of Research Use [14], is the extent to which potential adopters' intend to use and actually use the innovation in practice. Sustainability beyond the intervention depends on achieving positive outcomes at each of the patient, practitioner, and system levels, and the degree to which innovations are integrated into routine practices and organizational structures [15,16].

## Methods

**Figure 1:**
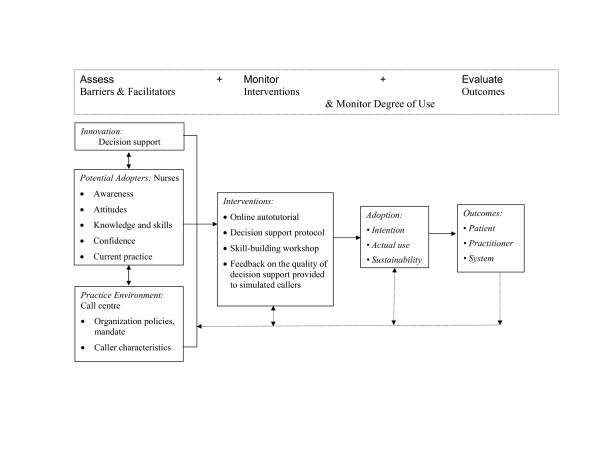
**Model of Implementation of Decision Support by Call Centre Nurses Adapted From the Ottawa Model of Research Use**. Note. From "Translating research: Innovations in knowledge transfer and continuity of care," by I.D. Graham and J. Logan, 2004, *Canadian Journal of Nursing Research, 36*, p. 94. Copyright 2004 by Canadian Journal of Nursing Research.

We conducted a theory-driven exploratory case study. The Ottawa Model of Research Use [14] guided the collection of qualitative and quantitative data and facilitated the triangulation across data sources (see Figure 1) [17,18]. Ethics approval was obtained from the Research Ethics Board at the University of Ottawa (#H 11-03-03).

### Participants and data sources

Table 1 summarizes the number and nature of the participants and data sources. Data collection methods included key informant interviews, focus groups, and a survey. Organizational documents gathered included monthly reports, minutes of meetings, organizational charts, newsletters, job descriptions, and advertisements informing the public of the program.

**Table 1:** Representativeness of data collected by source

Categories of participants	Nature of data sources	Number expected	Number participated
Purposeful sample of key informants: administrator setting strategic direction at the call centre, a nursing supervisor, a nurse educator, and a provincial ministry of health official	Individual interviews	4	4
Staff nurses exposed to the intervention	Focus groups	6 to 8	8
	Adoption of decision support protocol survey	31	25

### Data collection tools

The data collection tools (e.g., interview and focus group guides, survey) were designed to collect data on the adoption of the decision support protocol and factors influencing both the use of the protocol in practice and sustainable changes. These tools were based on the Ottawa Model of Research Use [14] and others used in a previous study of the baseline barriers to providing decision support [12,13].

Adoption of the decision support protocol was measured using a self-administered survey that included questions about whether or not the nurses had used the protocol and their intentions to use it in the future. Thirty-eight statements about factors that might influence the use of the protocol were rated on five-point Likert scales that ranged from *strongly agree *to *strongly disagree*. Questions in both the interview and focus group guides were grouped into the following categories: experiences using the decision support protocol; barriers and facilitators to using the protocol in practice; factors influencing the sustainability of nurses providing patient decision support; and ways to enhance how these types of calls are managed at the call centre.

### Analysis

The analysis, guided by the Ottawa Model of Research Use [14], focused on exploring answers to three main questions:

1. Was the decision support protocol adopted into clinical practice?

2. What effect did the intervention have on nurses' approaches to supporting real callers making values-sensitive decisions?

3. What factors are likely to influence the sustainability of values-sensitive decision support by call centre nurses?

Content analysis of the key informant interviews and focus groups transcripts was conducted to identify evidence to support each of these three questions. Common themes were inductively derived. Transcripts were analyzed using NVivo (version 2.0.163, QRS International Pty. Ltd.). Participants were sent summaries of the results from the interviews and focus groups in which they participated and were asked to verify their accuracy. Key organizational documents were analyzed to develop a rich description of the organization and to highlight concurrent activities that may have influenced the study.

Quantitative data were coded numerically and analyzed descriptively using SAS (version 8.01, SAS Institute Inc., Cary, NC, USA). Responses to the Likert scales in the survey were re-classified as agree (*strongly agree *or *agree*), disagree (*strongly disagree *or *disagree*), and neutral. To explore responses to each of the three main questions, the qualitative and quantitative findings from the multiple data sources were triangulated using NVivo.

## Results

### Characteristics of the participants and setting

The study took place at a Canadian province-wide health call centre serving a population of 4.2 million and averaging 22,600 calls per month. Of the 31 nurses who participated in the implementation intervention, 25 nurses (80.6%) responded to the survey and 8 participated in the focus groups (Table 1). The subset of nurses who completed the survey shared similar demographic characteristics with all those who participated in the intervention (Table 2). Four administrators (key informants) were also interviewed.

**Table 2:** Characteristics of the participants by data source

	Interviews & focus groups	Uptake survey	Implementation intervention
Frontline staff nurses	8 (66.7)	25 (100)	31 (100)
Nurse supervisor or educator	2 (16.7)	0	0
Non-nurse administrators	2 (16.7	0	0
Length of employment			
≤ 6 months	1 (8.3)	4 (16.0)	5 (16.1)
7 to 12 months	3 (25.0)	8 (32.0)	9 (29.0)
>12 months	8 (66.7)	13 (52.0)	17 (54.8)
Employment status (full-time equivalent)	Mean 0.77	Mean 0.75	Mean 0.74
not reported (casual status)	1 (8.3)	2 (8.0)	2 (6.5)
BSc or higher education	7 (58.3)	10 (40.0)	13 (41.9)
Gender			
Female	10 (83.3)	25 (100)	30 (96.8)
Male	2 (16.7)	0	1 (3.2)
Years of nursing			
≤ 5 years	0	0	0
6 to 10 years	0	1 (4.0)	2 (6.5)
11 to 15 years	1 (8.3)	7 (28.0)	7 (22.6)
≥ 16 years	9 (75.0)	17 (68.0)	22 (71.0)
not reported	2 (16.7)	0	0
***N***	12	25	31

Note: Data are numbers (%) unless otherwise specified

The call centre provides toll-free 24-hour telephone consultation by registered nurses to help residents manage their health and participate actively in making health decisions. Unique from other call centres in Canada [9], this call centre is part of an integrated self-care program that also provides public access to a self-care handbook and Internet-based health information resources, including over 95 patient decision aids. Monthly reports, from December 2003 to June 2004, indicated that about 55% of the calls concerned triaging symptoms, 25% were about a specific health condition, and 20% concerned other issues (e.g., drug information, finding health services). In 2003, the most common patient decision aids accessed by the nurses included those dealing with birth control methods, breast versus bottle feeding, male newborn circumcision, wisdom teeth removal, and treatment of miscarriage.

This call centre was established in April 2001 when the provincial ministry of health awarded a three-year contract to a private, not-for-profit management company. Of the 108 nurses employed in 2003, the typical nurse was female, had over 20 years of nursing experience, worked part-time hours, was unionized, and had worked at the call centre for one year or longer [12]. Nurses were grouped into three teams, each led by a nursing supervisor who reported directly to a non-nurse operations manager and indirectly to a nursing practice leader. The operations manager and nursing practice leader reported to a director of operations who reported to the provincial ministry of health.

Several mechanisms were in place to ensure program quality and minimize the risk of litigation [19,20]. On hiring, nurses received 105 hours of orientation and three months of mentoring. The orientation was focused mainly on triaging symptoms, with 0.75 hours devoted to introducing patient decision aids. The computerized protocols and health information database used to guide the telephone consultations were purchased from Healthwise^® ^Inc. and adapted for Canadian use. Call centre activities were monitored and reported to the provincial ministry of health on a monthly basis using a set of performance indicators (e.g., respond to 80% of calls within 20 seconds) based on the American Health Call Centre Accreditation Standards of the Utilization Review Accreditation Commission Inc. [21]. Monthly reports included statistics on call volume, call response time, call abandonment, length of calls, proportion of first time callers, call disposition (e.g., emergency, physician visit, self-care), pre- and post-call intent of the caller, and results of a quality audit on a random sample of audio-taped calls.

Over the study period from December 2003 to June 2004, there were several concurrent activities that were likely to have influenced this implementation study. In January 2004, nursing supervisors' roles and responsibilities were restructured. These changes resulted in the creation of a master staff development plan, major change in staffing patterns, and an expansion of call centre services (e.g., palliative care, newborn care). In March 2004, nurses started verifying caller demographics by linking to the provincial ministry of health's confidential database. Implementation of this practice change involved classroom training of all staff and subsequent performance review by nursing supervisors on real calls prior to autonomous practice. Over the study period, nurse absenteeism and inadequate staffing resulted in a higher number of calls in the hold queue and as a result, increased pressure for nurses to shorten their call length. Finally, the contract for the call centre services was due for renewal in the summer of 2004, which caused concern about job security among the nurses, increased organizational pressure to meet performance indicators, and re-directed administrative priorities to preparing a response to the imminently expected request for proposals.

### Was the decision support protocol adopted into clinical practice?

Eleven of the 25 nurses (44%) who participated in the implementation intervention (e.g., autotutorial, skill building workshop, decision support protocol, performance feedback) used the decision support protocol within one month following the intervention and the remaining 14 nurses (56%) reported that they had not received calls requiring values-sensitive decision support during that time. Twenty-one nurses (84%) agreed that they were comfortable using the decision support protocol. Most nurses (92%) indicated that they intended to use the protocol within the next three months. Nurses in the focus groups shared their experiences using the decision support protocol with real callers. One nurse spoke of the challenges of getting started and learning through her early experiences.

*It was just plunge in, see what you do the first time. So the first few I did, I did all in one day. And I may not have been right on all of them but I could see where I missed. The next one I thought was better*.

### What effect did the intervention have on nurses' approach to supporting real callers making values-sensitive decisions?

#### Recognize need for decision support

Nurses reported being more likely to recognize callers experiencing decisional conflict and highlighted the issues related to call classification. One nurse shared, "*Whereas before I might have asked a series of questions before I came to a realization that they were in a complex decision making process. Now I can identify much more readily*." Nurses identified that these calls would be difficult to identify in the database because they would usually be classified as a health condition-specific or medication-related call.

#### Improve decision support

Many nurses shared examples of how they thought their approach to providing decision support had improved. To exemplify, one nurse described how the protocol facilitated a more specific assessment: "*I'm more likely to ask questions about the decision and where they are on it instead of just making assumptions; which is a lot of what I did earlier*." Of the 25 nurses who completed the survey, over 90% agreed that the decision support protocol was logical (n = 23), helped prepare callers for discussing decisions with their practitioners (n = 24), complemented the nurses' usual approach (n = 23), and helped them to more fully explore the issues of importance to the callers (n = 24). All 25 nurses (100%) agreed that the new protocol facilitated caller empowerment. This was further supported by focus group nurses' description of callers being more engaged in the discussion; "*...and it's a dialogue and they really feel part of the dialogue*." Another nurse shared, "*...especially when you ask them the pros and the cons. You know suddenly the light goes on; like, I guess I could write them down*." Of the 25 nurses, 24 (96%) agreed that using the protocol provided a more consistent approach to supporting the callers. Several nurses described the new approach for handling these decision support calls as more efficient, streamlined, and shorter (e.g., "*with the specific tool to ask, I find the call goes quicker*").

#### Perceived practice changes positively

The importance of nurses providing patient decision support was supported by one nurse who said, "*Anybody can read the information the value of nursing in my philosophy is that you're helping counsel, guide. Provide information, yes, but not just a telephone operator*." Nurses also appreciated having a structured process for approaching these types of calls which took the pressure off having to find the 'right decision'. For example, "*I used to feel quite nervous that I felt like I should know the answer. So this has given me a lot of power that you can help them, that you don't have to sort it out for them*." Several nurses expressed their general satisfaction with their enhanced decision support role: "*For me, this is the most enjoyable part*"; "*I came out knowing I made a difference"; "That's the job I want to do, help people making any decisions*." Finally, some nurses reflected upon how these new skills in exploring values were relevant to symptom calls, particularly when callers did not agree with the triage decision determined by the protocol. For example, one nurse shared, "when we are sure they should call 911 and they're really reluctant and I always say well, what's the reason behind this so you kind of try to explore."

### What factors are likely to influence the sustainability of values-sensitive decision support by call centre nurses?

Barriers and facilitators influencing sustainability are presented in Table 3.

**Table 3:** Suggestions to enhance sustainability by overcoming barriers to nurses providing values-sensitive decision support

Most frequently identified barriers		Suggestions to enhance sustainability
*Innovation: Decision Support*	Patient decision aids are hard to use with patients over telephone	- Decision aids need more point form and auto-charting
	No structured process for preparing callers for shared decision making	- Resolved with use of Decision support protocol.
	Decision support protocol is not integrated with charting	- Integrate protocol in computer database with auto-charting ability
*Potential Adopters: Nurses*	Inadequate decision support knowledge	- Resolved by providing nurses with access to an autotutorial
	Inadequate skills in providing decision support	- Partially resolved with nurses participation in skill building workshop - Mentoring from supervisors to further develop nurses' skills - Revise call audit tool to include key decision support elements - Continuing education to reinforce learning - Encourage nurses to self-assess their performance
	Low confidence in ability to provide decision support	- Nurse supervisors could give positive feedback on quality of decision support provided
*Practice Environment: Call Centre*	Unclear program direction to provide decision support	- Determine impact of decision support calls on program services - Establish clear direction
	Limited orientation of new staff to decision support resources	- Use feedback to revise implementation intervention - Extend training to all nurses and in-particular nurse supervisors - Revise call audit tool to include elements of quality decision support
	Pressures to minimize call length	- Revise call classification to collect decision support calls statistics - Establish call length guidelines tailored to types of calls - Revise patient decision aids for easier use by telephone - Integrate decision support protocol into the database
	Low caller awareness that call centre nurses provide decision support	- Market decision support services to public & other health services

#### Decision support tools

To facilitate use of the decision support protocol and patient decision aids, nurses need to have these tools readily accessible for use over the telephone. In the survey, 18 nurses (72%) agreed that the protocol, in its current format as a word processing file, takes extra time to navigate, transfer into the documentation system, and use for documenting. By including the protocol as a screen within the documentation system, one nurse suggested, "...*as soon as you recognize that somebody is in one of these situations and you can push a button on your screen and have it pop in your call manager. How easy would that be? That would be swell!*" Nurses suggested that patient decision aids in the database needed to be easier to locate and revised for use over the telephone. For example, one nurse stated,

*And setting it up with pro's, con's, not big sentences to explain each point. I mean if we're supposed to know it, we're supposed to know it. So you know, you might want to have preambles for all this stuff, if you have to. But it is cut and dry. Get it short. Point form*.

As well, nurses wanted the protocol and the patient decision aids linked into the documentation system such that nurses' responses to questions would be automatically transferred into the electronic health record; similar to the way in which auto-charting occurs in symptom protocols.

#### Ongoing reinforcement for skill development

Nurses in the focus groups requested opportunities to support their applying these novel skills in practice. For example, "*it would be great just to have more of those simulated calls just to be able to do them*" and routine inservices focused on sharing experiences from decision support calls to offer a "*feeling of connection with other people who are doing them*". The nursing supervisors were identified as those best positioned to mentor the nurses, given their current responsibilities include providing feedback from call audits and coaching nurses to improve call handling. One nurse shared, "*If there is a problem with your times, what she *[nursing supervisor] *does is goes over that with you and tries to coach you and pulls calls that are long to see, you know, where you need shortening*." Nurses also expressed concern about a patient decision support call being randomly selected for the monthly call audit. "*I don't think that they *[nursing supervisors] *would know how to acknowledge what was done well and try to coach to what other things could be done better*." Although nursing supervisors were invited to participate in the intervention as non-study participants, competing demands due to organizational changes limited their availability to participate. Furthermore, their call audit tool did not include key elements necessary for quality patient decision support.

#### Fit of decision support with program direction

Nurses suggested that if supporting callers facing values-sensitive decisions is an expectation of their role this needed to be made clear in the program direction. To that end, appropriate changes would need to be made to organizational policies and procedures. Nine of 25 nurses (36%) felt that they had clear direction from the organization that they should be providing values-sensitive decision support. One administrator appeared less sure about the need for an organizational directive specific to providing patient decision support; "*...to communicate the value that this is a positive change for nursing practice as it takes the *[call centre] *in a new direction, in a direction I think we want to go in*". Administrator key informants identified that prior to an organizational commitment to having call centre nurses provide values-sensitive decision support they needed to determine the impact on call centre staffing, performance monitoring, the nursing education plan, and budget. Most nurses agreed that the call centre services should include patient decision support with 20 (80%) identifying all nurses and 2 (8%) identifying only a sub-group of specialized nurses as those who should be providing decision support guided by the new protocol.

#### Decision support training for other nurses

Of those surveyed, 22 nurses agreed (88%) that nurses would need education sessions, beyond their initial call centre orientation, to develop their knowledge and skills in values-sensitive decision support. In one focus group, a nurse suggested the need to "...*embed it in our continuing education program*". Our implementation intervention (i.e., decision support protocol, autotutorial, workshop, and performance feedback on simulated calls) was acceptable to over 90% of participants [13] and could be used for ongoing decision support training. Focus group nurses offered to be simulated patients for other staff developing these skills. The best timing for this type of training ranged from "*in their orientation week or the week after their orientation week so that they start out doing this when they're taking decision making type calls*" to three to six months after starting at the call centre.

#### Call length guidelines

Throughout the study, nurses were concerned that decision support calls would take longer than the organizational 12.5 minute call length target and requested that call length guidelines be tailored to types of calls. Despite this frequently identified barrier, one nurse in the focus group shared how she rationalized longer calls,

*...so I personally don't worry about it. And I find it all balances out...If you don't deal with it now, then it sort of goes down the line. It's going to take more time and money and everything else*.

Nurses also highlighted environmental pressures to minimize call length that included the flashing light on their telephone to indicate waiting calls, an electronic display board to indicate the number of callers waiting, and personal monthly reports on call length.

#### Marketing of decision support services

Administrators and nurses argued that, for sustainability, the public and health care providers needed to be informed about the decision support services available through the call centre. This was supported by the survey finding that only 4 of 25 nurses (16%) agreed that the public was aware that the call centre nurses could support people facing values-sensitive health decisions.

## Discussion

This is the first known study of the factors influencing adoption and sustainable implementation of values-sensitive decision support by call centre nurses. The selected call centre is unique in Canada because of its access to patient decision aids to support values-sensitive decisions. Yet the provision of decision support, using patient decision aids and nurse coaching, had not been fully implemented or evaluated. Our study demonstrated that the implementation intervention was successful in overcoming some barriers interfering with nurses' ability to provide quality values-sensitive decision support. The autotutorial and workshop facilitated nurses developing the knowledge and skills necessary for providing decision support and the decision support protocol provided nurses with a structured process to follow. Unaddressed barriers, particularly in the practice environment (e.g., pressure to minimize call length, protocol not integrated with the database, unclear program direction, and low public awareness), continue to interfere with nurses' adoption of the decision support protocol in practice. These barriers, if not managed, are likely to limit the sustainability of values-sensitive decision support services [14-16,22]. Moreover, without fully implementing these decision support services: (a) call centre nurses are likely to continue intervening by providing information only; (b) callers are likely to continue experiencing decisional conflict without making quality decisions; and as a consequence, (c) there may be deleterious effects on patient, practitioner, and health service outcomes [5,23-25]. The practice environment changes necessary to facilitate sustainable implementation of values-sensitive decision support are discussed below.

### Tailoring call length guidelines

In this and other studies time pressures have been found to negatively influence the implementation of decision support innovations [26-29]. In previous studies, the time pressures were due mostly to self-imposed time limits intended to limit waiting times for other patients. However, in this study nurses experienced organizational pressures to minimize call length resulting from call length guidelines, nurses' monthly feedback on their average call length, indicators of callers waiting, and inadequate staffing. At the same time the organization felt pressure to meet performance indicators and be considered favourably for contract renewal. In the absence of Canadian guidelines, performance indicators were based on American standards [21]. However, funding for healthcare in the US is organized differently than it is in Canada, with reimbursement from many American health plans dependant on members contacting call centres prior to using any other health services (including emergency departments) [9]. Therefore, standards that are congruent with the mandate of call centres within the Canadian healthcare context are needed.

Current pressure to minimize call length is likely to have a negative influence on quality of nursing worklife, recruitment of nurses to work at the call centre, absenteeism, and retention. Previous research on the psychosocial impact of call centre work found that call handlers reported poorer well-being and lower work-related satisfaction when their performance was constantly monitored [30]. Furthermore, nurses who are less satisfied with their work have higher levels of absenteeism and are more likely to leave their place of employment [31]. To facilitate nurses providing values-sensitive decision support without increasing their workload pressures, call centres providing these services could benefit from call length guidelines appropriately tailored for a variety of call types.

Recent evidence from values-sensitive decision support provided to patients face-to-face [6] and to simulated patients over the telephone [13], indicates that 18 to 20 minutes (plus time for collecting demographics and charting) may be a more reasonable call length target. There is the potential for more efficient use of time, if the decision support protocol were integrated into the computer database, the patient decision aids were revised for easier delivery via the telephone, and all of these tools were formatted for auto-charting.

### Including decision support in the program direction

Another important unresolved barrier is the lack of clarity in the call centre program direction. However, policy changes at the provincial ministry of health level that would encourage the provision of values-sensitive decision support by nurses in call centres are unlikely without evidence demonstrating the benefits of this service on patient and system outcomes. Prior to this study, evidence existed regarding the effectiveness of patient decision aids, [5] nurse decision support coaching, [6] and the feasibility of call centres for delivering values-sensitive decision support [12,13]. Based on feedback from nurses in the study, a plan to expand the decision support implementation intervention to all nurses at the call centre would need to start with the nursing supervisors. The nurse supervisors were described as those who: a) reinforce application of new knowledge and skills in practice; b) conduct the monthly audits of a random sample of calls; and c) provide feedback to nurses on performance issues.

Given the current call classification system, it would be challenging to monitor the volume of calls and the outcomes related to patient decision support quality. These calls are buried within health condition-specific and medication call data. One solution is to add a values-sensitive decision support call classification category to the database. Alternatively, integrating the decision support protocol within the database would facilitate tracking its use and help monitor individual callers' outcomes, such as changes in their progress through the stages of decision making, their decisional conflict, and their preferred option [32,33].

### Informing the public about decision support services

Decision support for people facing values-sensitive health decisions is not yet part of routine healthcare services and thus the public is not aware of how to get help with making these tougher health decisions. Strategies to inform the public about the call centre's services could explicitly include information about the availability of values-sensitive decision support from call centre nurses and patient decision aids within the programs' Internet-based health information resources. Alternately, client groups with unmet decisional needs (e.g., those deciding about birth control or major elective surgeries) could be targeted by marketing interventions either directly or by aligning the call centre with other healthcare services.

### Creating positive nursing workplace experiences

Nurses in the study were positive about their enhanced nursing practice after having used the decision support protocol. The study intervention helped nurses learn a generic process-driven approach to handling decision support calls. Although the process is generic, callers' values associated with options and the influence of others' opinions on their situation make most callers' situations unique. As well, nurses' responses confirmed that they believe decision support to be an important and personally valued part of their role. By providing values-sensitive decision support within their repertoire, nurses: (a) increase the diversity in their calls; (b) apply nursing expertise in novel ways; (c) use their nursing skills closer to full potential; and (d) receive feedback on individual caller outcomes such as progress in decision making. These characteristics of workplace activities have been demonstrated to improve quality of work-life and increase call centre nurses' satisfaction [29,30,34,35].

### Limitations

The strategies used to increase trustworthiness of the findings [17,36,37] in this study included theory guided analysis, triangulation of data sources, and participant verification of the interpretation of transcripts from interviews and focus groups. Despite these data collection and analysis strengths, the study has limitations. There was a potential for non-response bias and self-report bias in the survey. Although not all nurses responded to the survey, the demographic characteristics of the 25 nurses (80.6%) who responded were similar to those who had participated in the intervention (*n *= 31) (see Table 2). Furthermore, triangulation across data sources revealed consistent findings. Another limitation was the length of the evaluation. Longer-term evaluation of the adoption of the decision support protocol in nursing practice is warranted. Given the impending contract renewal, at the outset of the study there was clear direction that all study measures needed to be completed by June 2004.

## Conclusion

Call centre nurses in our study receive calls from people facing values-sensitive health decisions but several factors hinder the nurses from providing quality decision support. Following participation in an implementation intervention, nurses were more likely to adopt the decision support protocol in their telephone-based practice. Furthermore, nurses appreciated the shift from a content-driven to a process-driven approach to providing decision support, and had improved self-perception of their experiences with real callers. Nurses discussed using the protocol to guide these calls and better tailor their interventions to the assessed needs of callers. At the same time, the call centre organization became more sensitized to factors influencing nurses' approach to managing values-sensitive decision support calls.

However, unresolved barriers in the practice environment continued to interfere with implementing values-sensitive decision support and are likely to limit sustainability of nursing practice changes. For sustainability, nurses identified the need for clear program direction, the decision support protocol integrated in their documentation system, patient decision aids revised for easier use over the telephone, call length guidelines tailored to types of calls, decision support training provided for supervisors along with all staff, and marketing of these new services to the public.

## Competing interests

The author(s) declare that they have no competing interests.

## Authors' contributions

DS conceived the study, developed the protocol in collaboration with co-authors (AMO, IDG, MPP), recruited participants, collected the data, managed the data, carried out the statistical and qualitative analysis in collaboration with co-authors, drafted the manuscripts, re-drafted the manuscripts in collaboration with co-authors, and was responsible for the overall management of the study. Co-authors approved the final manuscript.
